# Nonspecific Cytotoxic Cell Antimicrobial Protein (NCAMP-1): A Novel Alarmin Ligand Identified in Zebrafish

**DOI:** 10.1371/journal.pone.0116576

**Published:** 2015-02-17

**Authors:** Margaret Mariscal Monette, Donald Lee Evans, Thomas Krunkosky, Alvin Camus, Liliana Jaso-Friedmann

**Affiliations:** 1 Department of Infectious Diseases, College of Veterinary Medicine, University of Georgia, Athens, Georgia, United States of America; 2 Department of Veterinary Biosciences and Diagnostic Imaging, College of Veterinary Medicine, University of Georgia, Athens, Georgia, United States of America; 3 Department of Pathology, College of Veterinary Medicine, University of Georgia, Athens, Georgia, United States of America; INRA, FRANCE

## Abstract

Cells from the coelomic cavity of adult zebrafish (zf) were used to study the alarmin-like activities of nonspecific cytotoxic cell antimicrobial protein-1 (NCAMP-1). Immunohistochemistry studies using polyclonal anti-NCAMP-1 identified constitutive NCAMP-1 in epithelial cells of the zf anterior kidney, in liver parenchyma and in the lamina propria of the intestine. NCAMP-1 was also located in the cytosol of mononuclear cells in these tissues. Cytosolic NCAMP-1 was detected in a diverse population of coelomic cells (CC) using confocal microscopy and polyclonal anti-NCAMP-1 staining. Large mononuclear and heterophil-like CC had intracellular NCAMP-1. These studies indicated that NCAMP-1 is constitutively found in epithelial cells and in ZFCC. To establish a relationship between NCAMP-1 and the alarmin functions of ATP, a stimulation-secretion model was initiated using zf coelomic cells (ZFCC). ZFCCs treated with the alarmin ATP secreted NCAMP-1 into culture supernatants. Treatment of ZFCC with either ATP or NCAMP-1 activated purinergic receptor induced pore formation detected by the ZFCC uptake of the dye YO-PRO-1. ATP induced YO-PRO-1 uptake was inhibited by antagonists oxidized-ATP, KN62, or CBB. These antagonists did not compete with NCAMP-1 induced YO-PRO-1 uptake. Binding of ZFCC by both ATP and NCAMP-1 produced an influx of Ca^2+^. Combined treatment of ZFCC with ATP and NCAMP-1 increased target cell cytotoxicity. Individually NCAMP-1 or ATP treatment did not produce target cell damage. Similar to ATP, NCAMP-1 activates cellular pore formation, calcium influx and cytotoxicity.

## Introduction

Alarmins are host-derived molecules that mediate inflammatory responses similar to those observed after binding of microbial products (Pathogen-Associated Molecular Patterns /PAMPs) to Toll-like receptors and other pattern recognition receptors (PRR) [1–2]. Although alarmins and PAMPs are derived from different sources, the host and pathogen, respectively, they are recognized by many of the same pattern recognition receptors (PRR). The term damage-associated molecular patterns (DAMPs) encompasses both alarmins and PAMPs and apply to those ligands causing damage or destruction of host cells [3]. Alarmins are rapidly released following cell necrosis but not during cellular apoptotic responses [2]. Under normal conditions, viable cells can secrete alarmins which can activate antigen presenting cells and may participate in homeostasis and wound healing by promoting tissue reconstruction [3, 4].

The majority of alarmins are preformed and can be rapidly released into the extracellular milieu. The best characterized alarmins are ATP, high mobility group box-1 (HMGB-1) and heat shock proteins [4–16]. HMGB-1 functions as a chemoattractant and activates antigen presentation following either passive release from necrotic cells or secretion by activated mononuclear cells [4, 8, 9]. HMGB-1 also mediates inflammatory responses by promiscuous binding to TLR4 and TLR9 and receptor for advanced glycation end products [6, 8, 11].

Similar to antimicrobial peptides including cathelicidin-derived LL37 peptide and defensins, most alarmins are found in the cytosol or in microvesicles. LL37 is cleaved from the parent cathelicidin molecule and as such activates innate immune responses by acting as a chemoattractant and inducing IL-1β secretion. One of the receptors implicated in the role of LL37 in inflammatory responses is the nucleotide purinergic receptor P2X_7_ [17–22]. Non-protein alarmins do not require enzyme activation, they occur in relatively large concentrations in the cytoplasm and include DNA, uric acid, and ATP. Once released, alarmins may induce release of proinflammatory cytokines and as well produce chemoattractant activity.

The most abundant tissue non-protein alarmin is ATP. Under normal conditions, ATP is present in the extracellular millieu in small concentrations but is rapidly released from a variety of cells under conditions of cell damage, hypoxia, ischemia, inflammation or even mechanical stress [23–28]. ATP binds to P2X_7_R. Although extensive work has been done to characterize functions of the P2X_7_R in humans and rodents, little is known regarding the function of this receptor in teleost species. One study found that endotoxin challenge led to a significant increase in macrophage IL-1β expression by gilthead seabream (*Sparus aurata*) [29–31]. The same investigators found that extracellular ATP promoted the release of IL-1β from a seabream fibroblast cell line (SAF-1 cells). This study indirectly supported the concept that the ATP receptor P2X_7_R may have participated in this response [30–31]. When the agonist BzATP was tested, permeabilization occured in a subset of acidophilic granulocytes and was completely reversible. Release of IL-1β did not occur from these cells after priming with ligands for different TLRs and NOD proteins as has been shown for ATP [31]. The zebrafish and seabream P2X_7_R were individually expressed in HEK293 cells to compare agonist and antagonist profiles to cells transfected with the rat P2X_7_ gene [32]. Responses to ATP and BzATP were greatly reduced in the cells expressing zebrafish and seabream P2X_7_R compared to the rat expressing cells.

A recent study characterized the cells lavaged from the coelomic cavity of adult zebrafish and found that diverse populations of functional immune cells including granulocytes, macrophages and monocytes, lymphocytes, and NCC (the teleost NK equivalent) were present [33]. This new source of cells in the zf provided a tissue in which alarmin binding to the P2X_7_R could be investigated. The zebrafish embryo has also been useful in studies of innate immunity such as the acute-phase response to infection and chemotactic responses to injury [34–35].

In the present study, the adult zebrafish was used as an animal model to investigate potential alarmin activity of NCAMP-1. NCAMP-1 (nonspecific cytotoxic cell cationic antimicrobial peptide-1) is expressed on the membranes of catfish NCC; it binds GpC and CpG oligodeoxynucleotides (ODNs); and similar to LL37 has direct antimicrobial activity [33–35]. In mammalian cells NCAMP-1 is expressed on the membrane of a human NK cell line (YT-INDY), it binds dGT20 and CpG ODNs [36]; it is found in granule extracts from RAW 264.7 cells; and NCAMP-1 is expressed by a variety of mouse leukocytes derived from the blood, mesenteric lymph nodes, and spleen [37]. The alarmin activity of NCAMP-1 has been reported in catfish cells [38]. Here, NCAMP-1 is compared to those effects induced by the established endogenous alarmin ATP. Both bind to cells harvested from the coelomic cavity of zebrafish; they induced pore-formation; and they upregulated the concentration of intracellular calcium. Both agonists also increased cell-mediated cytotoxicity of zebrafish cytotoxic cells. These data suggested that ATP and NCAMP-1 have alarmin activities but may initiate inflammation via different ligand-receptor activation pathways.

## Material and Methods

### Zebrafish care and maintenance

Adult WIK strain zebrafish were maintained at 81–82°F in a UV filtered fresh water flow through system at the University of Georgia Zebrafish facility.

### Ethics Statement

Permission for the use of zebrafish was granted by the University of Georgia Institutional Animal Care and Use Committee, and the specific permit number was: Animal Welfare Assurance #A3437–01. The University of Georgia is accredited by the Association for Assessment and Accreditation of Laboratory Animal Care, International. The PHS Assurance No. is A3437–01, and the expiration date is 11/30/2015. Animals were used to collect tissue samples and in vivo experiments were not conducted. The animals were ensured minimum suffering by maintaining the average water quality parameters at pH average of 7.2 and conductivity 450uS, using Instant Ocean to adjust. Fish were fed brine shrimp twice daily. Adult fish used in experiments ranged from 3 to 8 months of age and were semi-syngeneic by single pair brother-sister matings through the F6 generation. Euthanasia of zebrafish was accomplished by immersion in MS-222 (#TRS1; Aquatic Eco-Systems Inc., Apopka, FL) at 300ng/ml.

### Preparation of zebrafish coelomic cavity (ZFCC) cells

Female fish were euthanized with an overdose of MS222 and immediately intra-coelomic lavages were harvested into petri dishes containing 3ml trypsin-EDTA (Invitrogen, 15400–054) as previously described [33]. Briefly, the ZF were first cleaned with a dilute solution of ethanol, allowed to dry and ZF were injected into the coelomic cavity with sterile trypsin-EDTA using a 10 ml syringe and sterile 25 G polypropylene hub hypodermic needle (Kendall, 250321). A 10X solution of trypsin-EDTA (0.5% Trypsin and 0.2% EDTA in saline) was diluted to 1X concentration before use. Trypsin activity was inactivated with FCS after harvesting the cells. When indicated as ZFCC, the cells used for analysis were not gated and as shown before contained distinct populations of monocytes, lymphocytes, granulocytes, nonspecific cytotoxic cells and macrophages [38].

### Histology and IHC

Fish were euthanized with an overdose of MS222, and 10 minutes after cessation of movement the body cavity was injected with Dietrich’s solution. Total body fixation was accomplished by submersion into Dietrich’s solution [38]. An entire fish was placed into standard tissue cassettes for routine overnight processing. After the tissue was embedded in paraffin, 4 um-thick saggital sections were cut. Sections were deparaffinized and rehydrated through graded ethanols. Sections were stained with Gill's hematoxylin and eosin (H&E) using standard protocols. Adjacent sections were used for immunohistochemistry (IHC). Antigen retrieval was performed using a pressure cooker with citrate buffer (pH 6.0) for 10 min at 120°C. Power Block (Biogenex, San Ramon, CA) was applied to all sections for 5 min. Rabbit polyclonal anti-NCAMP-1 antibody was diluted 1:5,000 in 50mM Tris-buffered saline (TBS) (pH 7.4). Normal rabbit IgG was also diluted 1:5,000 in TBS. Diluted primary antibodies were applied to sections and incubated for 60 min at room temp. After washes, a biotinylated anti-rabbit IgG (Biogenex, HK340–9K, San Ramon, CA), a Super Sensitive Multilink, was applied to all sections for 15 min. Next, sections were incubated with an alkaline phosphatase label conjugated to streptavidin (Biogenex, HK 331–9K, San Ramon CA) for 15 min. Lastly, the sections were incubated with Vulcan Fast Red chromogen (Biocare Medical, FR805, Concord, CA) for 5 min.

### Intracellular NCAMP-1 staining

ZFCC were harvested from adult females and were fixed with 4% paraformaldehyde in PBS with 0.1% NaN_3_ (PBSN) for 20 min rt. ZFCC were then permeabilized with 0.1% saponin in PBSN (PBSNS) for 15 min. After 2x5min washes [once with PBSN and once with BBS with 0.1% NaN_3_ and 0.1% Saponin (BBSNS)] cells were incubated for 1hr with either polyclonal rabbit anti-NCAMP-1 antibody (1–2ug) or normal rabbit IgG (isotype control) at identical concentrations. Samples were washed (BBSNS and PBSNS) and suspended in goat anti-rabbit IgG conjugate, Texas red (Invitrogen, #), in PBSNS for 1h. Samples were washed (PBSN) for flow cytometric analysis. A conjugate control was included in the flow analysis. Nonspecific staining (percent fluorescence isotype control) was subtracted from the anti-NCAMP-1 binding to provide positive NCAMP-1 staining.

### Immunofluorescence microscopy

Eight well chamber slides (Lab-Tek II, Nunc) were coated with 0.01% poly-L-lysine (37°C, 1h), cells were added to the wells (30 min/room temperature), and fixed with 4% paraformaldehyde in PBS with 0.1% NaN_3_ (PBSN/20 min). Cells were washed 2x5min, permeabilized with 0.1% Saponin (in PBSN for 20min) and incubated with Image-iT signal enhancer (Invitrogen, Carlsbad, CA, US) for 30min. Slides were washed with PBSN and 0.1% Saponin (PBSNS), blocked with 10% normal goat serum in BBSNS for 15 min, and incubated with either polyclonal rabbit anti-NCAMP-1 or normal rabbit IgG (isotype control) for 60 min. Sequentially slides were washed again, once with PBSNS and once with PBSNS containing goat anti-rabbit IgG Texas Red-conjugate and treated with phalloidin-FITC [25μg/ml (Sigma) 30 min]. Cells were washed and the preparation was mounted with Prolong Gold Anti-fade and DAPI (Invitrogen, P36935). Images were captured with a Zeiss Axiovert microscope, using a 63X 1.4NA objective and images analyzed with Axiovision software.

### Western blot analysis of soluble NCAMP-1

ZFCC were harvested from female fish, counted and suspended in non-FCS containing RPMI media. Cells were either treated with 5mM ATP or media. After 30 min the supernatant was collected for analysis. Each sample was TCA precipitated (20%) on ice for 1h. After several washes with ice cold acetone, SDS was added to each sample. Recombinant NCAMP-1 was run as a positive control and prepared as previously described [39]. Samples were run on 11% SDS gels and transferred to nitrocellulose. Filters were blocked for 15 min at room temperature (Superblock, Pierce Rockford, IL, USA, #37545, with 0.05% Tween 20). Primary antibodies included rabbit polyclonal anti-NCAMP-1 [33, 39] and purified normal rabbit IgG (NRbIgG) as an isotype control. Primary antibodies were diluted in Borate Buffered Saline (pH 8.5) supplemented with 1% BSA, and 0.05% Tween 20. Peroxidase-conjugated goat anti-RbIgG secondary antibody (Pierce, 31463, 1:50,000 dilution) was diluted in Super blocking buffer. Blots were developed using ECL (Pierce Supersignal West Pico, 1856135).

### Ligand binding to ZFCC

ZFCC were harvested from female zf, counted, and suspended in cold PBS with 0.1% NaN_3_ and 1.0% BSA (PAB/60 min/4°C). ZFCC (2 x 10^5^) cells were incubated with 0.5 μg of recombinant Cy3-NCAMP-1, washed once with PAB and analyzed by flow cytometry. Inhibition of fluorescence was determined by pretreatment of ZFCC with 0.5 μg of cold (non-Cy3 labeled) NCAMP-1.

### Agonist induced YO-PRO-1 iodide uptake

ZFCC were harvested and suspended in RPMI at room temperature. ZFCC (100,000cells/100ul) were suspended in 5μM YO-PRO-1 iodide (Invitrogen, Y3603). Recombinant NCAMP-was expressed and purified as previously described [39] and diluted in 10 mM phosphate buffer (pH 8.0). At indicated concentrations, soluble NCAMP-1 was added to the cell suspension and immediately analyzed for fluorescence as compared to YO-PRO-1 uptake in the absence of NCAMP-1. The same protocol was carried out with varying concentrations of ATP (Sigma). Toxicity of treatment (e.g. necrosis) was measured by addition of 2μg/ml PI (Invitrogen).

### Calcium mobilization assay

Harvested ZFCC were washed with RPMI and suspended in RPMI at 1x10^6^/ml. Cells were loaded with 4μM Fluo-4 AM (Invitrogen, F-1217) in the presence of Pluronic F-127 (Molecular Probes, Eugene, OR, USA, F-127), a high affinity calcium indicator, for 20min at 37°C. The cells were washed in media and resuspended at room temperature for analysis of mean fluorescence intensity (MFI) by flow cytometry. Dye-loaded cells were analyzed for 50 seconds to generate a baseline at which time either calcium ionophore (A23187), 1μg NCAMP-1 or indicated concentrations of ATP were added and analyzed immediately. Continued ZFCC containing media and dye were the negative controls at 50 sec.

### Inhibition of ATP induced activity

Inhibition of ATP/NCAMP-1 induced effects on ZFCC activity was done by pretreatment with KN-62 ((*S*)-5-Isoquinolinesulfonic acid 4-[2-[(5-isoquinolinylsulfonyl)methylamino]-3-oxo-3-(4-phenyl-1-piperazinyl)propyl]phenyl ester, 1-[N,O-bis(5-Isoquinolinesulfonyl)-N-methyl-L-tyrosyl]-4-phenylpiperazine, Sigma, I2142), Comassie Brilliant Blue (CBB), or oxidized-ATP (oATP produced by the 2’- and 3’-hydroxyl moieties oxidized to aldehydes by periodate treatment) (Sigma). Before analysis of ligand binding, YO-PRO-1 or Fluo-3 were added and pore formation or Ca^2+^ influx was measured as described above.

### Cytotoxicity

Cytotoxicity was measured using a carboxyfluorescein succinimidyl ester (CFSE)-based nonradiometric flow cytometric assay [33]. Human promyelocytic leukemia cells HL-60 (ATCC CCL 240) were used as target cells (TC) while ZFCC served as effector cells (EC). HL-60 targets were suspended at 5mM CFSE (Sigma, 21888) in sterile PBS for 15min at 37°C. Cells were washed and suspended at 5 x 10^4^ cells per 100μl. Before co-incubation, zebrafish EC were treated with 1μg NCAMP-1, 5mM ATP or media for 30min at 37°C, then washed oncewith media. Zebrafish EC and TC were combined at an effector:target ratio of 8:1. Control samples contained only CFSE labeled target cells and media. Flow analysis was conducted at 0, 1, 2, and 4hrs co-incubation at 37°C and 5% CO_2_. Cytotoxicity was quantified by measuring the time (45s counts) and volume dependent loss of CFSE labeled targets (in the target cell gate) in the presence of unlabeled zf effector cells.

### Flow cytometry

Flow cytometry was performed with two instruments and the results are shown as non-gated ZFCC. Analysis of YO-PRO-1 uptake and cytotoxicity assays was done using an EPICS XL-MCL four color analysis system (Beckman-Coulter Electronic Corp) equipped with a 15 mW air cooled argon-ion laser operating at 488nm wavelength. Data collection analysis was performed using Coulter System II software version 3.0. ZFCC extracellular ligand staining and calcium influx assays were done using an LSRII (Becton Dickinson) equipped with a solid state 488nm coherent sapphire blue laser (50mW). Data collection analysis was performed using Facs DIVA software version X and FlowJo software version 7.5.

### Statistical analysis

Where indicated results are shown as the mean +/- standard error of 3 independent experiments. Statistical significance was calculated by two-way ANOVA, according to the Bonferroni method or by Duncan’s (New) Multiple Range Test.

## Results

### NCAMP-1 is detected in different zebrafish tissues

**Fig 1:**
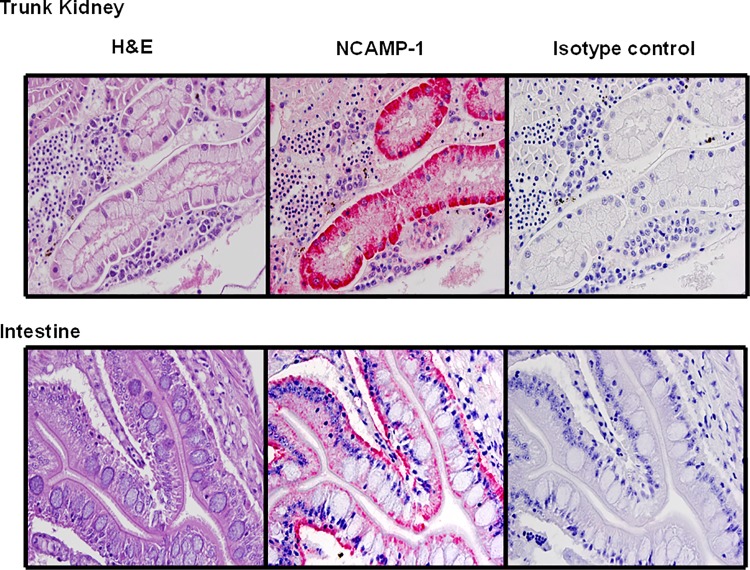
Constitutive expression of NCAMP-1 in zebrafish tissues. Serial thin sections of zf trunk kidney and intestine were examined by H&E staining; rabbit polyclonal anti-NCAMP-1 serum and normal rabbit isotype control.

To demonstrate *in vivo* expression of NCAMP-1 in zebrafish, whole adult females were fixed, sectioned, and mounted onto one slide. Histopathological evaluations were performed on a section of an entire fish. Serial sections of individual fish were alternatively stained using H&E (Fig. 1). Sections were also stained with a rabbit polyclonal anti-NCAMP-1 antibody (Fig. 1) and a normal rabbit IgG antibody (isotype control) (Fig. 1) to observe tissue localization of NCAMP-1. Positive staining for NCAMP-1 occurred in the head kidney along renal venules (the isotype control was negative) (Fig. 1, upper panel). Intense staining was seen by cells lining the endothelium of the entering arterioles. Positive staining for NCAMP-1 in the liver was less intense and more diffuse compared to the staining of the kidney. Intense staining was seen in macrophage-like cell dense areas (negative isotype controls). Staining in the intestine (Fig. 1, lower panel) occurred in the luminar surface of the epithelial cells and in the lamina propria.

### Intracellular NCAMP-1 expression in cells of the zebrafish coelomic cavity

**Fig 2:**
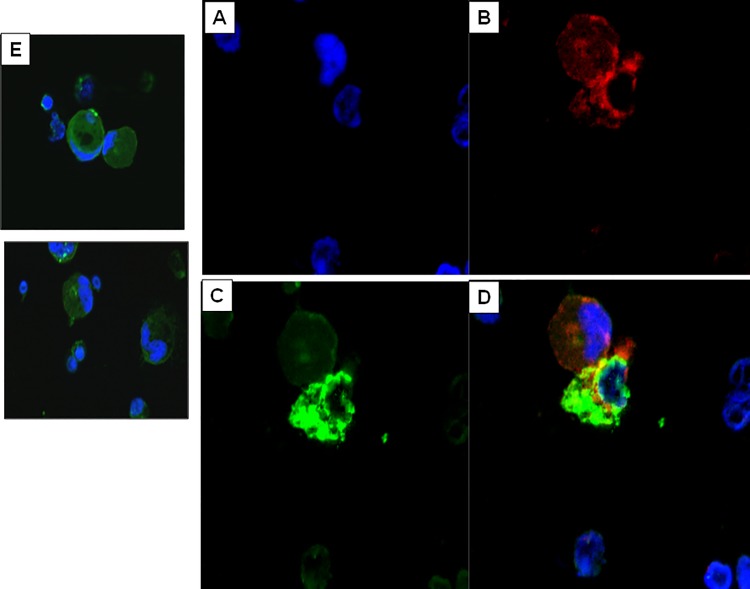
Intracellular NCAMP-1 expression in zebrafish coelomic cells (ZFCC). ZFCC were fixed, permeabilzed and stained for intracellular NCAMP-1 (red) with rabbit anti-NCAMP-1 polyclonal antibody or normal rabbit IgG. Actin was stained with phalloidin (green) and nuclear morphology is indicated with DAPI staining (blue). (A) is DAPI only; (B) anti-NCAMP-1 only; (C) actin; and (D) are the merged images. E shows the negative isotype controls (DAPI + isotype control).

We previously showed by flow cytometry that NCAMP-1 is expressed on the membrane of different populations of ZFCC [33]. Here, intracellular expression of NCAMP-1 in zebrafish coelomic cavity cells (CC) was detected with polyclonal anti-NCAMP-1 by confocal microscopy Fig. 2 A-E). Single color images are shown in Fig. 2A, 2B and 2C with nuclear staining DAPI (blue), NCAMP-1 (red) and Actin (green), respectively Isotype controls were negative (Fig. 2E). Positive punctate staining of NCAMP-1 (Fig. 2D) was observed in a variety of cells including macrophages, heterophils and lymphocyte-like cells.

### NCAMP-1 is secreted by ZFCC in response to alarmin ATP

**Fig 3:**
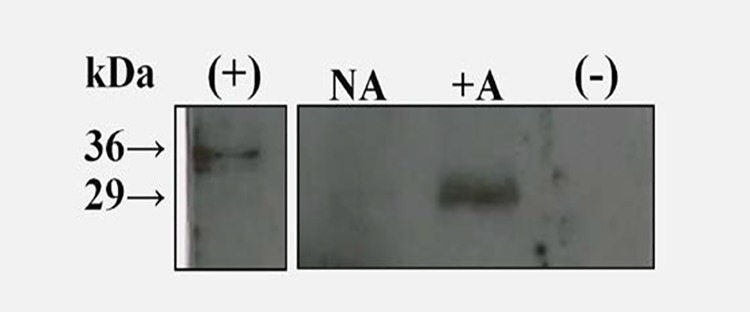
Western blot analysis of NCAMP-1 release from ATP treated zebrafish CC. ZFCC were untreated (NA) or treated for 30 min with 5 mM ATP (+A). Supernatants were harvested and analyzed by Western blot using rabbit polyclonal anti-NCAMP-1 antibody. (+) is the positive control of R-NCAMP-1-Histag binding. (-) is the negative control (e.g. no primary antibody).

To determine if NCAMP-1 was released by ZFCC following *in vitro* treatment with ATP, supernatants from treated cells were collected after 30 min incubation with 5 mM ATP (or media control). Western blot analysis by polyclonal anti-NCAMP-1 showed a 29 kDa band in the gel containing the supernatant from ATP treated cells (Fig. 3). The molecular weight discrepancy between the secreted and recombinant NCAMP-1 forms has been previously noted in catfish [38]. The very basic nature of the protein is partly responsible for inaccuracies in SDS gel electrophoresis migration.

### Soluble NCAMP-1 binds to ZFCC and binding is inhibited by cold NCAMP-1

**Fig 4:**
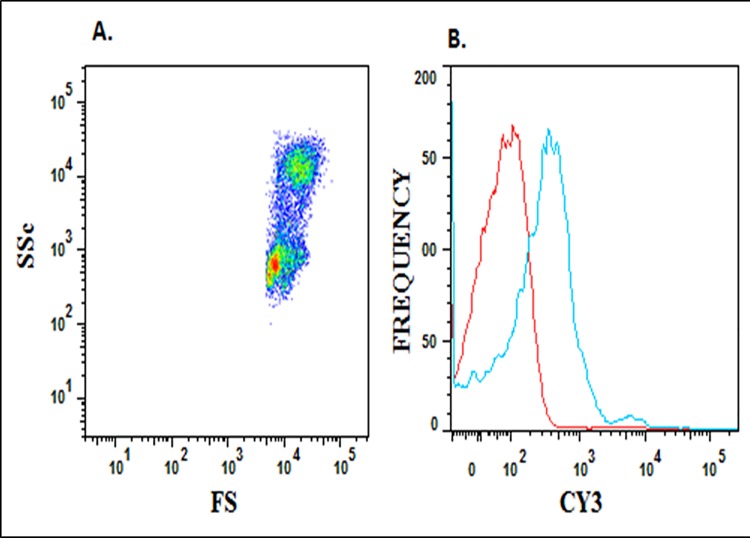
Binding of NCAMP-1 to ZFCC. Zebrafish coelomic cells were analyzed by flow cytometry in a two parameter histogram for their forward scatter and side scatter characteristics (PanelA). Panel B shows the ungated cells stained with 0.5 μg of Cy-3-NCAMP-1. The red histogram is the media (negative) control.

ZFCC were analyzed by flow cytometry and incubated with recombinant Cy3-labeled NCAMP-1 t in the presence and absence of unlabeled recombinant protein. Fig. 4A shows the forward scatter and side scatter histogram of the ZFCC populations analyzed for fluorescence in Fig. 4B. The one parameter histogram of the binding of Cy-3- rNCAMP-1 shows optimal staining at 0.5μg rNCAMP-1 with 50% cells positive. Addition of equal concentrations of cold-NCAMP-1 (0.5 μg) decreased binding to 30% and 1 μg decreased staining to 19%.

### P2X7 pore formation is induced by binding of NCAMP-1 or ATP as measured by YO-PRO-1 uptake

**Fig 5:**
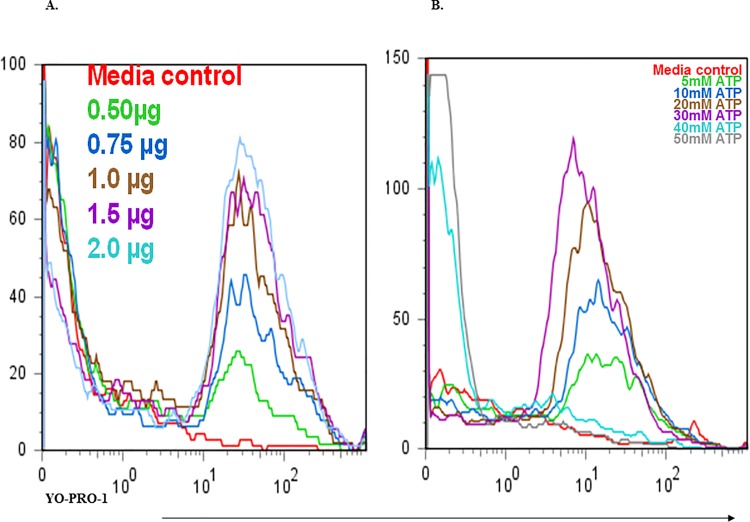
Soluble NCAMP-1 and ATP bind to ZFCC and induce a concentration dependent uptake of YO-PRO-1 dye. Indicated concentrations of soluble NCAMP-1 (A) or ATP (B) were individually added to a suspension of ZFCC at 5mM YO-PRO-1 and immediately analyzed by flow cytometry. Histograms are representative of three independent experiments per agonist at each concentration.

Soluble rNCAMP-1 (Fig. 5A) or ATP (Fig. 5B) were added to ZFCC in the presence of 5μM YO-PRO-1. Dye fluorescence was measured within seconds after addition of either agonist. Optimal concentrations of Cy3-rNCAMP-1 (1μg) produced 20–25% YO-PRO-1 fluorescence compared to 5% fluorescence for media control. ATP induced YO-PRO-1 uptake occurred with 30% fluorescence produced at 30 mM ATP concentration.

### ATP addition in combination with rNCAMP-1 does not induce additional YO-PRO-1 uptake

To observe the effects of multiple agonists on YO-PRO-1 dye uptake by ZFCC, rNCAMP-1 was added to ZFCC in the presence of 5uM YO-PRO-1 alone or followed by addition of increasing concentrations of ATP. In the presence of both agonists, neither additive nor synergistic effects on YO-PRO-1 staining occurred (Table 1). The results in Table 1 show that addition of NCAMP-1 did not significantly change the YO-PRO-1 uptake by ATP at any of the concentrations tested.

**Table 1:** YO-PRO-1 Uptake by ZFCC in response to NCAMP-1 treatment in the absence or presence of ATP.

ZFCC TREATMENT		% YO-PRO POSITIVE CELLS
		(Mean ±S. E.)
MEDIA		9.55 ± 3.88
NCAMP-1		14.7 ± 3.15
ATP (5mM)		16.2 ± 5.45
ATP (5Mm) +NCAMP-1		20.8 ± 2.02
ATP (10mM)		20.5 ± 7.11
ATP (10mM) +NCAMP-1		26.5 ± 3.41
ATP (20mM)		27.7 ± 8.8
ATP (20mM) +NCAMP-1		29.23 ± 6.32
ATP (30mM)		36.9 ± 8.69
ATP (30mM) +NCAMP-1		38.8 ± 7.21*

* P = ≤ 0.05 Different from NCAMP-1 only; Duncan’s (new) Multiple Range Test.

### Effects of P2X_7_R antagonists on ATP induction of YO-PRO-1 uptake

**Fig 6:**
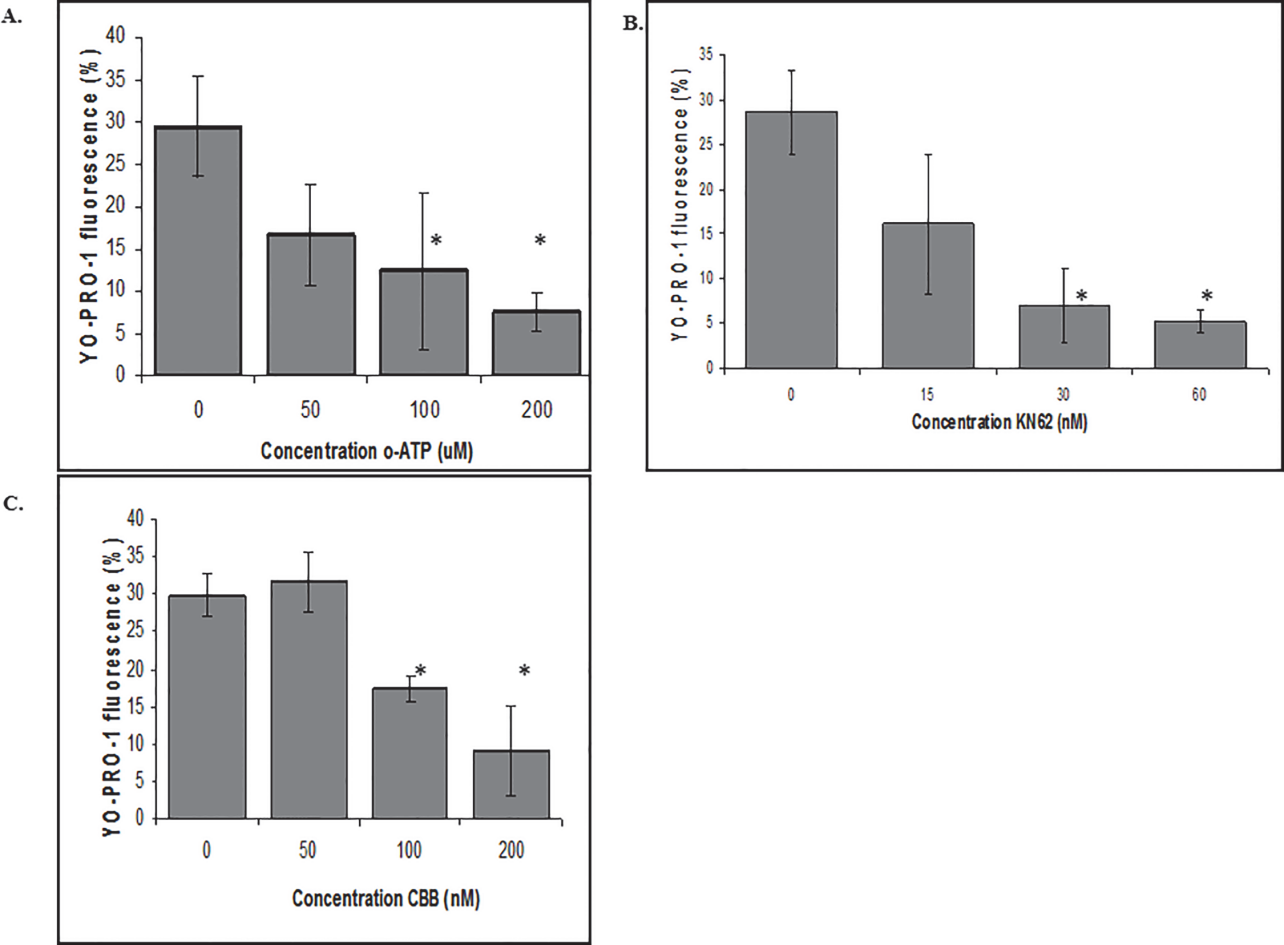
ATP induced YO-PRO-1 uptake by ZFCC is abrogated with P2X_7_R inhibitors. 30mM ATP was added to ZFCC with 5mM YO-PRO-1 and analyzed for percent fluorescence immediately or incubated with oxidized-ATP (oATP) (A), KN62 (B), or CBB (C) for 15 minutes prior to addition of ATP. Results are shown as the mean of 3 independent experiments ± S.E. * P≤ 0.01 significantly different from media controls as calculated by two-way ANOVA according to Bonferroni method.

To determine whether blockage of P2X_7_R activation of pore formation was linked to ATP treatment, three different ATP antagonists were tested: oxidized-ATP (oATP), Commassie Brilliant Blue G (CBB), and KN62. Prior to exposure to 30mM ATP, ZFCC were incubated for 15 minutes with oATP (Fig. 6A), KN62 (Fig. 6B), or CBB (Fig. 6C), and analyzed for YO-PRO-1 uptake. Each inhibitor abrogated fluorescence induced by ATP in a concentration dependent manner compared to controls. CBB and KN62 required low nanomolar concentrations for inhibition whereas oATP inhibited at micromolar concentrations. Similar experiments to determine the effects of receptor antagonist pretreatment and rNCAMP-1 induced YO-PRO-1 uptake were negative.

### Agonist induced YO-PRO-1 uptake is not due to cell death

**Fig 7:**
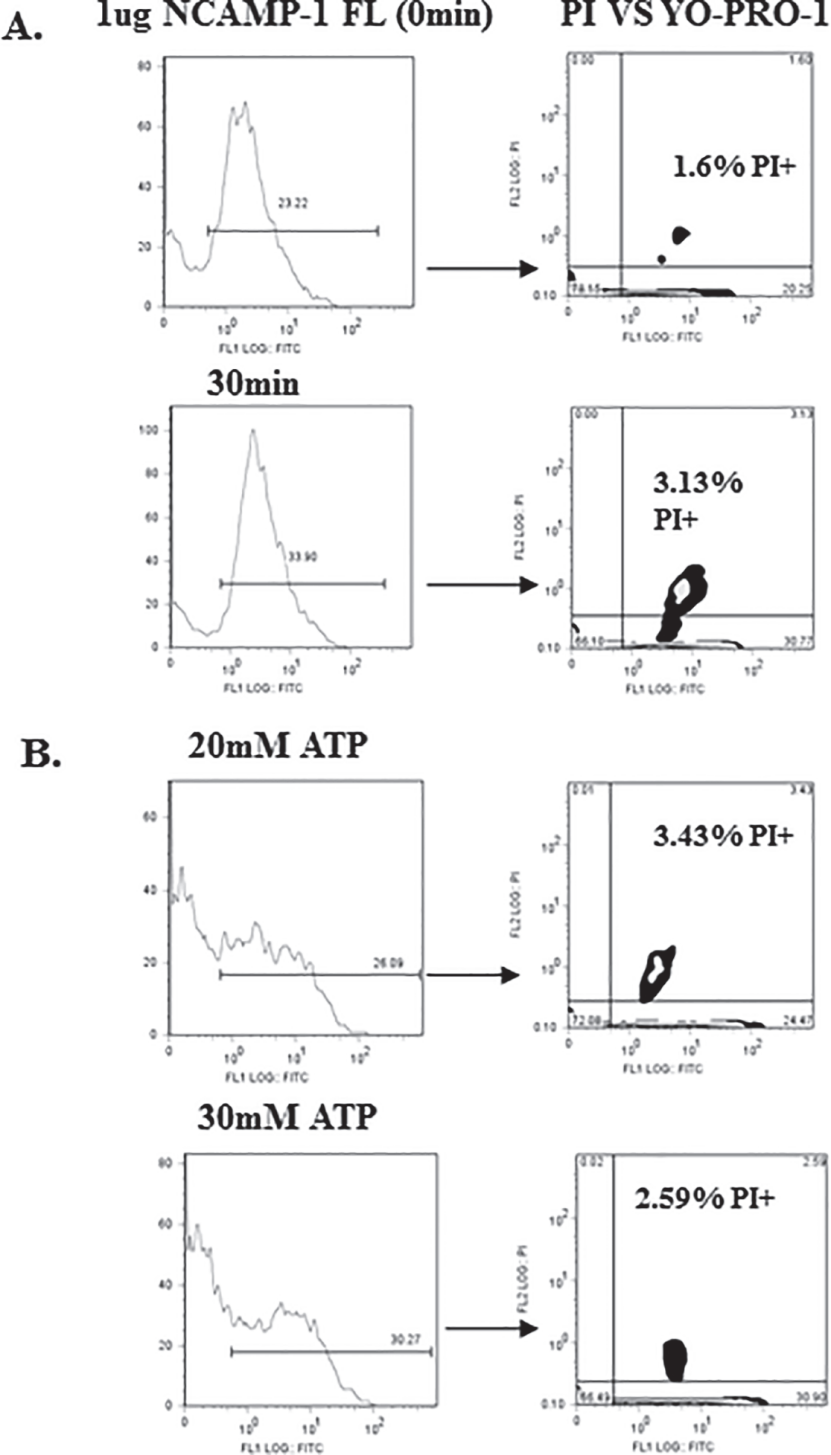
NCAMP-1 or ATP do not cause cellular damage. (A) ZFCC were incubated with 1ug NCAMP-1 for 30min at room temperature and analyzed with YO-PRO-1 and PI. (B) 20mM and 30mM ATP were added in presence of YO-PRO-1 and PI and immediately analyzed (0min). Representative of 3 independent experiments.

To determine whether ATP and NCAMP-1 induced pore-formation was produced by nonspecific toxicity, cells were simultaneously stained with propidium iodide (PI) and YO-PRO-1. Only 3–4% of the cells were PI+ (Fig. 7A). YO-PRO-1 uptake induced by NCAMP-1 was not due to cellular damage. Immediate exposure to ATP (Fig. 7B) resulted in approximately <5% PI positive cells.

### Intracellular Ca^2+^ influx is induced by rNCAMP-1 or ATP

**Fig 8:**
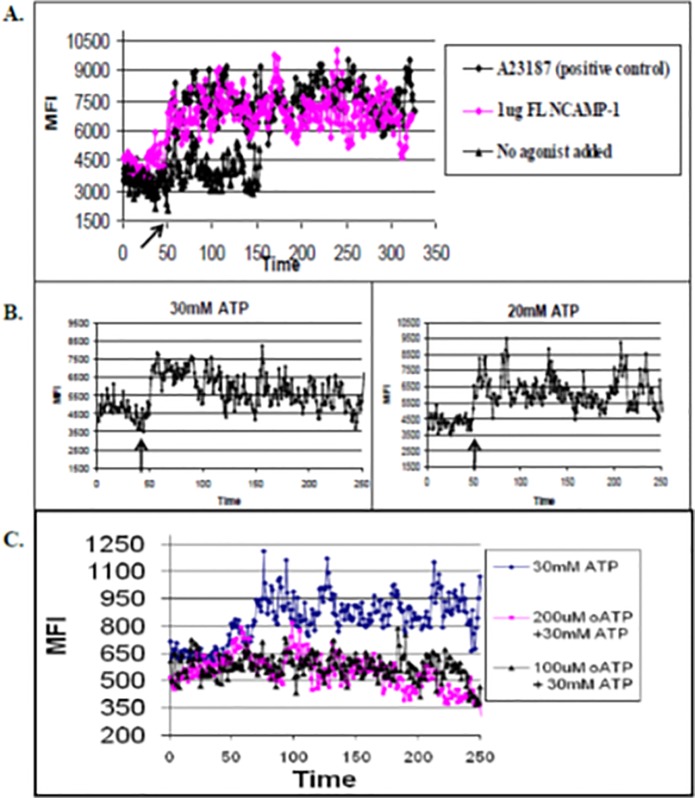
NCAMP-1 and ATP bind to CC and induce an influx of Ca^2+^ ions. ZFCC were loaded with 4 uM Fluo-4 for 20 minutes at 37°C, washed with media, then analyzed for mean fluorescence intensity by flow cytometry. 50 seconds into analysis the calcium ionophore (positive control) or 1ug NCAMP-1 (A) or different concentrations of ATP (B) were added and analysis was continued. In C, cells were treated with the indicated ATP antagonist before addition of 30mM ATP and analyzed. Arrows indicate the addition of the agonist. Representative of 3 independent experiments.

To determine whether rNCAMP-1 induced intracellular calcium mobilization, ZFCC were loaded with 4 μM Fluo-4. To establish a baseline for cellular constitutive calcium levels, rNCAMP-1, the Ca ionophore A23187, or media controls (Fig. 8A) were added and following 50 seconds Fluo-4 loaded cells were analyzed. Similar to the effect observed with the calcium ionophore (A23187), rNCAMP-1 induced a two fold increase in (Mean Fluorescence Intensity) MFI compared to the media control. ATP produced a similar calcium signal at 20 and 30 mM ATP (Fig. 8B). Results indicated that similar to the positive control, rNCAMP-1 and ATP induced a calcium influx in ZFCC. The effects of ATP antagonists on ATP induced calcium flux were measured. In Fig. 8C. ZFCC were loaded with Fluo-4 and then treated with two different concentrations (100μM or 200μM) of the antagonist oxidized ATP (oATP). After 50 seconds treatment, 30 mM ATP was added to each preparation (arrow) and cells were immediately analyzed by flow cytometry to determine free calcium levels (Fig. 8C). Inhibition was observed at both inhibitor concentrations (100 and 200 μM oATP).

### Pre-treatment of zebrafish cytotoxic cells with rNCAMP-1 or ATP increased cell-mediated killing of target (HL-60) cells

**Fig 9:**
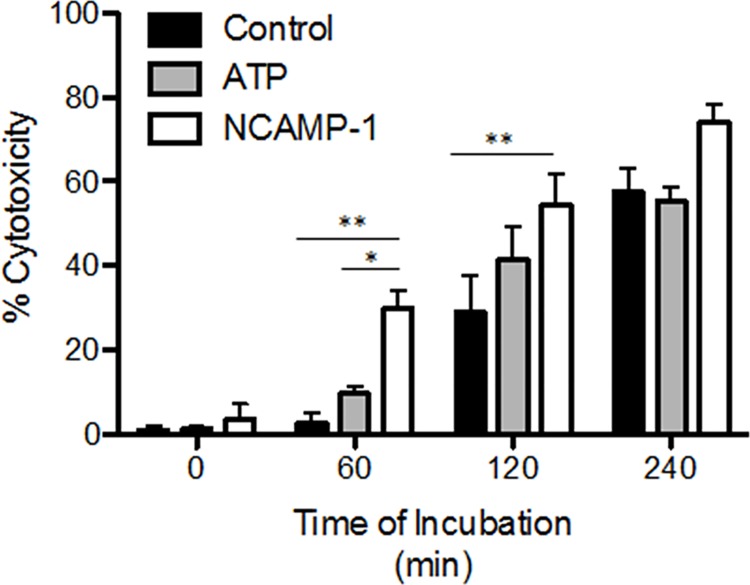
Cytotoxic activity of zebrafish effectors is increased following pretreatment of NCAMP-1 or ATP. HL-60 target cells were labeled with 5mM CFSE for 15 minutes at 37°C and washed. Targets were added to zebrafish effectors, pretreated for 30 minutes with either 5mM ATP, 1ug NCAMP-1, or no treatment, at an effector to target cell ratio of 8:1. Flow analysis was conducted at times 0, 1, 2, and 4 hours of co-incubation. Data points represent the mean ± standard error of 3 independent experiments. At times of 60 and 120 minutes, significant differences were observed between NCAMP-1 and media (** P≤ 0.01); NCAMP-1 and ATP *P≤ 0.05 as calculated by two-way ANOVA according to Bonferroni method.

The effect(s) of rNCAMP-1 and ATP on the cytotoxic effector function of ZFCC were next determined. A CFSE based cell-mediated cytotoxicity assay was done as previously described [33]. ZFCC (effector cells) were treated with rNCAMP-1 before co-incubation with CFSE labeled HL-60 target cells for 0, 60, 120, and 240 minutes post-treatment at an effector target cell ratio of 8:1 (Fig. 9). Significant increases in cytotoxicity were observed after 60 and 120 minutes in the lysis of target cells incubated with effectors treated with ATP or NCAMP-1. ATP pretreatment produced a similar effect as rNCAMP-1 at 120 minutes of co-incubation.

## Discussion

The present research is the first to study alarmins in a model system using coelomic cells from the adult zebrafish (*Danio rerio*). The coelomic cavity contains representatives of all the cells of the peripheral blood of fish (and mammals) in addition to macrophages and other cells with morphologies similar to undifferentiated cells of the mammalian bone marrow [33]. Although it has been reported that zf have a similar immune system to humans, the adult zf has been considered as an unlikely source of sufficient numbers of cells to use in *ex vivo* immune studies [31]. Immunohistochemistry and functional analysis have identified characteristic nuclear morphologies of leukocytes which were harvested in enough numbers to test their functions such as phagocytosis and cytotoxicity suggesting that that zf coelomic cells (ZFCC) may be used *in vitro* to develop a zf model. NCAMP-1 was first identified [40] from catfish nonspecific cytotoxic cells (e.g. NCC). The purified and subsequently sequenced protein [nonspecific cytotoxic cell cationic antimicrobial protein (e.g. NCAMP-1)] is a novel phylogenetically conserved H1X-like protein [38, 40]. We now show similarities and distinctions between the alarmin functions of NCAMP-1 and ATP, and we suggest that NCAMP-1 shares similarities also with other alarmins such as high mobility group box-1/HMGB-1 [7–8]. HMGB-1 and NCAMP-1 are both non-microbial cationic proteins of similar molecular weights [9–10]; both are expressed endogenously by many different types of cells (e.g. endothelial, epithelial) (Figs. 1–2) and are released by microbial, mechanical or chemical damage to cells [38]. Also similar to HMGB-1, NCAMP-1 is constitutively found in “normal” fish serum and is upregulated following bacterial infections [38]. We predict that similar to HMGB-1, histone-1X-like NCAMP-1 may also bind to DNA. Unlike HMGB-1 however, the previously described antimicrobial killing activity of NCAMP-1 is similar to that of another alarmin, cathelicidin LL-37 [4–46]. Taken together these studies strongly suggest that NCAMP-1should be categorized along with ATP, HGMB-1, and LL37 into a family of alarmins with the unique characteristic of sharing some similarities but combining most of their properties into a single molecule.

In the present study comparisons were made between NCAMP-1 and ATP. Activation of the P2X_7_R has not been described in any teleost *ex vivo* experiment. In transfection experiments of cDNA from gilthead seabream (*Sparus Aurata*) and zebrafish P2X_7_R into HEK 293 cells, responses to ATP and BzATP were greatly reduced compared to cells expressing the rat transcript [29]. Endotoxin challenge produced a significant increase in IL-1β expression in seabream macrophages [30]. The same investigators found that consistent with stimulation of the P2X_7_R, treatment with endotoxin and ATP promoted the release of IL-1β from a seabream fibroblast cell line (SAF-1 cells) [31]. This did not occur in the presence of different TLRs and NOD proteins [30,31]. Comparisons of those results with the present study is difficult as our inhibitor data was conducted *ex vivo* on teleost cells and strongly suggested that NCAMP-1 and ATP induce pore formation and YO-PRO-1 uptake. It is presently unknown whether ATP and NCAMP-1 bind to a common and/or different receptors. We have previously demonstrated in catfish anterior kidney cells a strong link between NCAMP-1 and cellular as well as inflammasome activation of inflammatory caspase-1 activity [38]. These data are suggestive that these two molecules are minimally capable of initiation of release of IL-1β. Further studies are required to establish the linkage between NCAMP-1 binding and the molecular properties of the inflammasome activities of ZFCC.

The ability of ATP to induce cellular pore formation (e.g. demonstrated by the uptake of YO-PRO-1) was first observed in mammalian peritoneal mast cells [47]. Currently the ATP outer membrane receptor is more commonly studied in macrophages and lymphocytes. YO-PRO-1 only enters cells by purinergic receptor specific pores [23–24]. The properties of ATP-evoked YO-PRO-1 uptake closely resemble those of ATP-evoked ionic currents [24]. Addition of BzATP produced reversible dye and ion currents are similar to studies using ATP [25]. This function of P2X_7_R activation has also been measured in cells transfected with rat P2X_7_R [26] and human P2X_7_R [48]. In contrast to the study using HEK 293 cells transfected with zebrafish P2X_7_R [31], the present research demonstrated that ATP induced pore-formation was detected by YO-PRO-1 uptake (Fig. 5B). Soluble full-length recombinant NCAMP-1 induced pore-formation in ZFCC in a concentration dependent manner (Fig. 5A). 30 mM ATP produced 30% fluorescence in ZFCC compared to a range of 18–25% YO-PRO-1 positive cells following treatment with 1μg NCAMP-1. Secretion of IL-1β was not determined, so it is unknown whether LPS, or another PAMP, is necessary as a co-stimulus [49]. As previously suggested, it did not appear that LPS priming was necessary to induce pore-formation in ZFCC by ATP or NCAMP-1.

All of the ATP-induced activities observed in this study were blocked by ATP antagonists (Fig. 6). YO-PRO-1 uptake was blocked in a concentration-dependent manner by oATP, KN62, and CBB (Fig. 6A, B, and C, respectively). NCAMP-1 induced activity, however, was not blocked by any of these inhibitors, at any concentration. These findings suggested that NCAMP-1 may bind to a different site on the P2X_7_R than ATP, or NCAMP-1 may bind to a different receptor than ATP.

ATP-induced processing and release of IL1β was reported to coincide with cell death [50] and cell lysis has been suggested as the mechanism of release of IL1β [50]. In the present study, propidium iodide (PI) was used in the YO-PRO-1 uptake assays to ensure observations were derived from viable cells (Fig. 7) [51,52]. Immediate analysis of short term exposure of ZFCC to ATP indicated that pore-formation did not initiate cell lysis (Fig. 7B) and was not damaging to cells. No more than 4% fluorescence of PI occurred in cells exposed to 20 and 30 mM ATP. However, long term exposure (1hr) to 30 mM ATP did induce high percentages of PI fluorescence. The production of calcium influx as well as phospholipase activation is also characteristic of P2X_7_R activation. This activity is thought to be partially responsible for the secretion of IL-1β. Although IL-1β secretion from zebrafish immune cells was not determined in the present study, similar to calcium ionophore A23187, treatment of ZFCC with both 20–30 mM ATP and as well as soluble NCAMP-1 induced an approximate 2-fold increase in Ca^2+^ influx (Fig. 8).

The human cathelicidin-derived peptide LL37, originally described as a potent antimicrobial peptide, is capable of caspase-1 activation and stimulation of IL-1β [53] via the P2X_7_R in LPS-primed monocytes. Furthermore, P2X_7_R inhibitors, KN04, KN62 and oATP, suppressed pore-formation as measured by inhibition of YO-PRO-1 uptake and IL-1β release by mouse macrophages [54]. It is important to note that LL37 is also involved in chemotactic activity mediated by the formyl peptide receptor family (formyl peptide receptor-like 1, FPRL1) [20]. As an endogenous danger signal it is not well understood how NCAMP-1 is released from immune cells but it is important to demonstrate that this occurred *ex vivo* from viable zf cells. In view of its antimicrobial activity, it cannot be ruled out that NCAMP-1 may be released from PMNs as part of the formation of neutrophil extracellular traps (NETs) which have been described in fish [55–56]. The involvement of NCAMP-1 in NETs is presently not known.

Finally, cell-mediated cytotoxic activity was reported in cells harvested from the coelomic cavity of zebrafish [33]. This immune function could be upregulated by inflammatory stimuli. Incubation with low concentrations of ATP and NCAMP-1 prevented nonspecific ZFCC toxicity. However cytotoxic activity of ZFCC against target HL-60 cells was increased (Fig. 9) following treatment with ATP or NCAMP-1 compared to media controls. NCAMP-1 treated cells, had higher percent cytotoxicity at lower E:T ratios and at shorter co-incubation time points compared to ATP and media treated cells.

In the present study we demonstrated that populations of cells exist within the zebrafish coelomic cavity such that sufficient numbers can be harvested for analysis of several different types of cells. The distribution of cell types and expression levels of NCAMP-1 in ZFCC have clearly been demonstrated. Similar to DAMP activation, it appears that NCAMP-1 acts as an endogenous protein that may interact with other DAMPs (or indeed PAMPs) to activate various cellular activities. Whether NCAMP-1 enters cells by multiple membrane binding events such as receptor activation, facilitated transport, pinocytosis, endocytosis, etc. it is clear that interaction with the purinergic receptor can now be included in that repertoire of binding events. Future studies are required to investigate this interaction at signaling and transcription levels.
